# Prescription of antimicrobials in primary health care as a marker to identify people living with undiagnosed HIV infection, Denmark, 1998 to 2016

**DOI:** 10.2807/1560-7917.ES.2019.24.41.1900225

**Published:** 2019-10-10

**Authors:** Raquel Martin-Iguacel, Court Pedersen, Josep M Llibre, Jens Søndergaard, Frederik Veitland Ilkjær, Janne Jensen, Niels Obel, Isik Somuncu Johansen, Line Dahlerup Rasmussen

**Affiliations:** 1 Department of Infectious Diseases, Odense University Hospital, Odense, Denmark; 2 Infectious Diseases Department and Fight AIDS Foundation, University Hospital Germans Trias i Pujol, Badalona, Barcelona, Spain; 3 University of Southern Denmark, Department of Public Health. The Research Unit of General Practice, Odense, Denmark; 4 Department of Internal Medicine, Kolding Hospital, Kolding, Denmark; 5 Department of Infectious Diseases, Copenhagen University Hospital, Rigshospitalet, Copenhagen, Denmark

**Keywords:** HIV diagnosis, late HIV presentation, antibiotic use, antimicrobial use, missed opportunities, HIV testing, indicator conditions

## Abstract

**Background:**

Development of additional diagnostic strategies for earlier HIV diagnosis are needed as approximately 50% of newly diagnosed HIV-infected individuals continue to present late for HIV care.

**Aim:**

We aimed to analyse antimicrobial consumption in the 3 years preceding HIV diagnosis, assess whether there was a higher consumption in those diagnosed with HIV compared with matched controls and whether the level of consumption was associated with the risk of HIV infection.

**Methods:**

We conducted a nested case–control study, identifying all individuals (n = 2,784 cases) diagnosed with HIV in Denmark from 1998 to 2016 and 13 age-and sex-matched population controls per case (n = 36,192 controls) from national registers. Antimicrobial drug consumption was estimated as defined daily doses per person-year. We used conditional logistic regression to compute odds ratios and 95% confidence intervals.

**Results:**

In the 3 years preceding an HIV diagnosis, we observed more frequent and higher consumption of antimicrobial drugs in cases compared with controls, with 72.4% vs 46.3% having had at least one prescription (p < 0.001). For all antimicrobial classes, the association between consumption and risk of subsequent HIV diagnosis was statistically significant (p < 0.01). The association was stronger with higher consumption and with shorter time to HIV diagnosis.

**Conclusion:**

HIV-infected individuals have a significantly higher use of antimicrobial drugs in the 3 years preceding HIV diagnosis than controls. Prescription of antimicrobial drugs in primary healthcare could be an opportunity to consider proactive HIV testing. Further studies need to identify optimal prescription cut-offs that could endorse its inclusion in public health policies.

## Introduction

Despite extensive efforts in healthcare policies, ca 50% of new HIV cases in Europe continue to be diagnosed at late stages of the disease (CD4^+^ T-cell count < 350 cells/μL) and ca 30% present with very late HIV infection (CD4^+^ T-cell count < 200 cells/μL or presenting with an AIDS-defining illness (ADI)) [1-3].

Late presentation is associated with higher rates of morbidity and mortality, poorer response to antiretroviral therapy (ART), incomplete immune recovery, increased healthcare costs and increased risk of ongoing HIV transmission [4-11]. Therefore, late diagnosis has negative consequences for both the individual and society as a whole, some of which are irreversible. In the Collaboration of Observational HIV Epidemiological Research Europe (COHERE) study, late HIV diagnosis was associated with a 6–13-fold increased risk of ADI and/or death in the first year after HIV diagnosis, depending on the area in Europe [12]. Furthermore, the Strategic Timing of Antiretroviral Treatment (START) study showed that early diagnosis and ART initiation (at CD4^+^ T-cell count > 500 cells/μL) was associated with lower rates of serious ADI and of non-ADI including death [7]. Initiation of ART in individuals with very high CD4^+^ T-cell counts (> 750 cells/μL) has also been associated with a reduction in rates of ADI, especially malignant ADI [4]. Taken together, there is a large body of evidence supporting the benefits of identifying HIV infection at earlier stages.

A number of medical conditions (so-called indicator conditions) have been associated with an undiagnosed HIV prevalence > 0.1% and data from the United States (US) and France have shown that HIV testing in conditions above this threshold is cost-effective [13-15]. European guidelines recommend routine HIV testing in individuals presenting with indicator conditions or an ADI, in high-risk groups or in conditions where not identifying HIV would adversely affect the management of the disease [16-19]. Furthermore, guidelines in the United Kingdom (UK) recommend universal HIV testing for all adults presenting for care in any healthcare setting in geographical areas with high HIV prevalence, defined as > 0.2% [20]. Despite these recommendations, many missed opportunities for earlier HIV diagnosis occur in both primary and secondary healthcare [21-23].

Denmark has achieved the target from the Joint United Nations Programme on HIV/AIDS (UNAIDS) of 90% diagnosis, treatment and undetectability [24]. However, ca half of the infected individuals are still diagnosed at late stages of the HIV infection and therefore, new strategies to deal with this problem are needed.

With data from the Danish HIV Cohort Study, we recently found that HIV-infected individuals have frequent contacts with primary healthcare (PHC) in the 3 years preceding their HIV diagnosis. Consequently, PHC could play a major role in earlier detection of occult HIV infection [25].

Antimicrobial prescription in PHC is related to the treatment of some of the infectious ADI or indicator conditions such as bacterial infections (recurrent pneumonia, sexually transmitted infections (STI)), viral infections (herpes simplex, herpes zoster), and fungal infections (candidiasis), and its prescription could be regarded as a proxy for these conditions. Therefore, the aim of the present study was (i) to assess antimicrobial drug prescription (i.e. antibiotics, antivirals, and antifungals) in the 3 years preceding the HIV diagnosis compared with age and sex-matched controls, and (ii) to assess whether consumption of antimicrobials was associated with an increased risk of subsequent HIV diagnosis.

## Methods

### Setting

On 31 December 2017, Denmark had a population of 5.7 million with an estimated HIV prevalence among the adult population of 0.1% [26].

The Danish healthcare system is universally tax-funded, guaranteeing free access to healthcare for all citizens in Denmark. Antimicrobials can only be obtained with a prescription from a physician in primary or secondary care or administered during hospitalisation.

### Study period

The study was performed from 1 January 1998 to 31 December 2016.

### Data sources

We used the Danish personal identification number, a unique 10-digit personal identifier assigned to all Danish citizens at birth or upon immigration, to link individuals in the following national healthcare registries:

### The Danish HIV Cohort Study

The Danish HIV Cohort Study (DHCS) is an ongoing nationwide, prospective, population-based cohort study of all HIV-infected patients treated at Danish hospitals since 1 January 1995. The cohort has been described in detail elsewhere [27]. The DHCS consecutively enrols patients newly diagnosed with HIV and immigrants with HIV infection. Data are updated yearly.

### The Danish Civil Registration System

The Danish Civil Registration System (DCRS), established in 1968, is a national registry, which stores information on vital status, residency and migration for all Danish residents. It is updated daily (Monday through Friday). All immigrants registered as living in Denmark are included. While undocumented migrants have access to healthcare through a temporary registration number, they cannot be followed over time through the administrative registries [28]. 

### The Danish National Prescription Register

The Danish National Prescription Register (DNPR) collects individual-level data on all medical prescriptions redeemed at Danish community pharmacies since 1 January 1995. The registry includes variables related to the patient, prescriber, pharmacy and dispensed drug. The latter includes date of dispensing, name, drug code, anatomical therapeutic chemical classification (ATC) code (available to 5th ATC level), number of packages, package size, strength, number of defined daily doses (DDD) per package, and drug formulation. The registry does not contain data on drugs used during hospital admission [29].

### Design

The study was designed as a case–control study nested in the DHCS.

### Study population

#### Cases

From the DHCS, we identified all adults (≥ 18 years) in Denmark diagnosed with HIV infection between 1 January 1998 and 31 December 2016 who had been living in Denmark in the 3 years before their HIV diagnosis. All cases were stratified as (i) very late HIV diagnosis (VLHIV), i.e. CD4^+^ T-cell count < 200 cells/μL or presentation with an ADI within 6 months of the HIV diagnosis, regardless of the CD4^+^ T-cell count, (ii) late HIV diagnosis (LHIV), i.e. CD4^+^ T-cell count 200–349 cells/μL, and (iii) early HIV diagnosis (EHIV), i.e. CD4^+^ T-count ≥ 350 cells/μL or documented seroconversion within the last 12 months, regardless of CD4^+^ T-cell count. Study inclusion was the date of HIV diagnosis.

#### Population controls

For every HIV-infected individual, we identified 13 age and sex-matched population controls from the DCRS. We chose this number of controls to secure the statistical power of the analysis. None of the controls had been diagnosed with HIV before 31 December 2016. All controls had to be alive and living in Denmark in the 3 years before HIV diagnosis for the matched case. Study inclusion was the date of HIV diagnosis for the matched case.

### Exposure

Using data from the DNPR, we assessed antimicrobial consumption based on all redeemed prescriptions of oral antimicrobial drugs in the 3 years before study inclusion. The consumption was further expressed as DDD per person-year (PY) (for names and ATC codes see Supplementary Table S1). Only those drug classes prescribed to > 1% of the HIV-infected individuals were included for further analysis (beta-lactams, macrolides, fluoroquinolones, tetracycline, antibiotics only used to treat urinary tract infections (UTI drugs), antivirals, azoles and nystatin).

For each antimicrobial drug class, we categorised the consumption based on different DDD cut-offs. These cut-offs were chosen based on clinical criteria as the average estimated number of DDD for one standard treatment for the most common infections (Supplementary Table S1).

Consumption of each antimicrobial class was assessed according to (i) the total DDD/3 PY, in the whole 3-year study period before HIV diagnosis and (ii) the percentage of patients in each DDD/PY category both per year and in the whole 3-year period.

### Statistics

For all antimicrobial drug classes, we assessed the fraction of cases and controls in each drug consumption group (i.e. category of DDD/PY) as defined above.

To identify exposures associated with risk of subsequent HIV diagnosis, we used conditional logistic regression analysis to compare exposure to different levels of antimicrobial drug consumption between cases and controls, providing an odds ratio (OR) and a 95% confidence interval (CI). These exposures were estimated both as categorical (DDD categories defined above vs no consumption) and as binary variables (consumption above different DDD/PY cut-offs vs consumption at or under this level). Based on the rare disease assumption, these OR were used as estimates of the relative risk (RR) of acquiring HIV.

To analyse the trend of increase in consumption of antimicrobial drugs in the different years before HIV diagnosis, we used the Cochran–Armitage test.

As there was a clinical and statistically significant interaction between the person’s sex and the risk of HIV diagnosis (likelihood ratio test; p < 0.01), we further stratified the analyses by sex. In a sensitivity analysis, we further stratified the data by HIV subgroup (EHIV, LHIV and VLHIV), mode of infection, Danish origin and age group (18–39, 40–59 and ≥ 60 years).

Finally, based on the assumption that we would test for HIV all exposed individuals within certain cut-offs for antimicrobial consumption, we used the fraction of exposed controls and cases in the study to estimate the percentage of people being tested, the percentage being diagnosed and the effectiveness factor [25]. The effectiveness factor was calculated by dividing the fraction of cases that would be diagnosed by the fraction of controls that would be tested and provided an estimation of how effective the interventions at different cut-offs would be compared with testing at random in a population with the same demographical pattern as our sample. Only a factor > 1 was considered effective.

STATA software (version 14) was used for data analysis.

### Ethical statement

The study was approved by the Danish Data Protection Agency (journal no 2008–41–1781). Ethics approval and individual consent are not required by Danish legislation governing this type of research on HIV-infected individuals.

## Results

### Baseline characteristics

**Figure 1:**
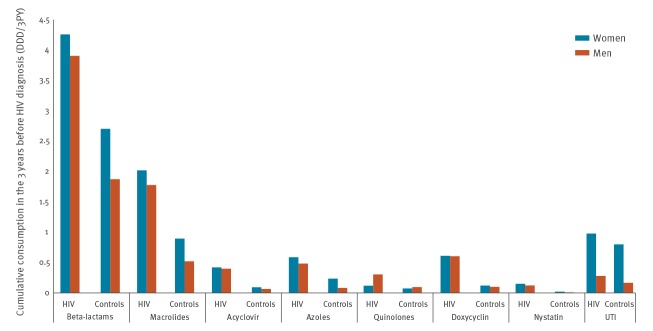
Antimicrobial drug prescription in the 3 years before HIV diagnosis, stratified by sex, Denmark, 1998–2016 (n = 38,976)

We identified 2,784 cases and 36,192 controls that fulfilled the inclusion criteria in the period from 1998 to 2016. Eighty per cent of cases and controls were male and the median age at diagnosis was 39 years (interquartile range (IQR): 32–48). Sexual transmission was the main infection route among cases, heterosexual for women (80%) and homosexual for men (61%). The majority of cases and controls were of Danish origin (male cases: 81%, male controls 86%, female controls 85%), except female cases of whom only 49% were of Danish origin. Non-Danish origin, older age and heterosexual transmission route were more common among those presenting with LHIV and VLHIV compared with EHIV. Although a slight decrease in the fraction of VLHIV was observed in recent calendar years, the fraction of LHIV and VLHIV remained high during the period 2010 to 2016 (16.9% and 30.7%, respectively). Table 1 shows additional baseline characteristics of the study population. During the 3 years before diagnosis, 72.4% of cases and 46.3% of controls had at least one prescription with any of the antimicrobial drugs included in the analysis (p < 0.001) (Table 1). Beta-lactams and macrolides were the two most frequently prescribed antimicrobial drug classes for both cases and controls in the study period. For all antimicrobial drug classes, we found a substantially higher prescription rate in cases than controls. Despite the overall higher prescription for women compared with men, this trend was independent of sex (Figure 1).

**Table 1:** Baseline characteristics of the study population, Denmark, 1998–2016 (n = 38,976)

Characteristics	HIV n = 2,784		Controls n = 36,192		HIV subgroups					
					VLHIV n = 954		LHIV n = 481		EHIV n = 1,349	
	n	%	n	%	n	%	n	%	n	%
Male	2,237	80.4	29,081	80.4	744	78.0	372	77.3	1,121	83.1
Age at HIV diagnosis, median years (IQR)	39 (32–48)		39 (32–48)		43 (36–52)		39 (32–47)		36 (30–44)	
**Age at study inclusion (years)**										
18–39	1,478	53.1	19,231	53.1	370	38.8	255	53.0	853	63.2
40–49	727	26.1	9,431	26.1	299	31.3	128	26.6	300	22.2
50–59	381	13.7	4,962	13.7	183	19.2	63	13.1	135	10.0
** **≥ 60 years	198	7.1	2,568	7.1	102	10.7	35	7.3	61	4.5
**Danish origin^a^**										
Yes	2,075	74.5	31,064	85.8	671	70.3	340	70.7	1,064	78.9
No	709	25.5	5,128	14.2	283	29.7	141	29.3	285	21.1
**Infection mode**										
MSM	1,365	49.0	Not recorded		366	38.4	230	47.8	769	57.0
Heterosexually infected	1,029	37.0	Not recorded		442	46.3	178	37.0	409	30.3
PWID	191	6.9	Not recorded		41	4.3	31	6.4	119	8.8
Other/unknown	199	7.2	Not recorded		105	11.0	42	8.7	52	3.9
HCV	267	9.6	Not recorded		77	8.1	38	7.9	152	11.3
Hepatitis B core antibody-positive	397	14.3	Not recorded		163	17.1	79	16.4	155	11.5
CD4^+^ T-cell count at study, median cells/μL (IQR)	330 (130–540)		Not recorded		73 (30–140)		276 (236–310)		541 (430–690)	
Viral load at study inclusion, median log_10_ copies/mL (IQR)	4.8 (4.1–5.4)		Not applicable		5.2 (4.8–5.9)		4.7 (4.2–5.2)		4.5 (3.7–5.0)	
**Calendar year of diagnosis^b^**										
1998–2003	825	29.6	Not applicable		349	42.3	135	16.4	341	41.3
2004–2009	1,168	42.0	Not applicable		362	31.0	212	18.2	594	50.9
2010–2016	791	28.4	Not applicable		243	30.7	134	16.9	414	52.3
**Antimicrobial prescription in the study period^c^**	2,015	72.4	16,750	46.3	743	77.9	324	67.4	948	70.3
Antibiotics	1,895	68.1	15,973	44.1	688	72.1	304	63.2	903	66.9
Antiviral	353	12.7	736	2.0	172	18.0	55	11.4	126	9.3
Antifungals	382	13.7	1,563	4.3	223	23.4	45	9.4	114	8.5

EHIV: early HIV diagnosis; HCV: hepatitis C virus; HIV: human immunodeficiency virus; IQR: interquartile range; LHIV: late HIV diagnosis; MSM: men who have sex with men; PWID: people who inject drugs; VLHIV: very late HIV diagnosis.

^a^ p < 0.01, Pearson’s chi-squared test.

^b^ Presented as row percentages.

^c^ One or more prescriptions of at least one of the antimicrobials included in the study.

DDD/3PY: defined daily dose per person in the 3-year study period; HIV: human immunodeficiency virus; UTI: urinary tract infection.

### Analysis by antimicrobial drug prescription levels

**Figure 2:**
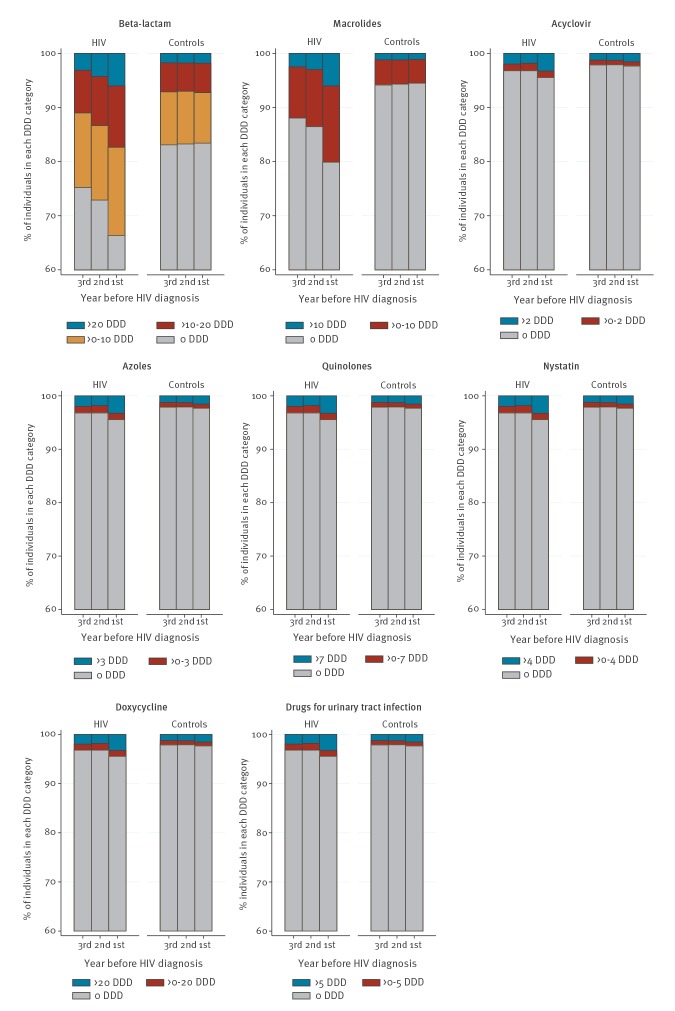
Prescription of antimicrobials for cases and controls, by year before HIV diagnosis of the matched case, Denmark, 1998–2016 (n = 38,976)

DDD: defined daily dose; HIV: human immunodeficiency virus; UTI: urinary tract infection.

For all analysed antimicrobial drugs, we found a significantly higher fraction of cases than controls with a high drug prescription based on the predefined DDD levels per PY. While drug prescription in HIV cases increased substantially the closer the date was to the HIV diagnosis, it remained stable among controls throughout the 3 years (Figure 2 and Table 2). This difference was statistically significant for beta-lactams, macrolides, quinolones, acyclovir, azoles and nystatin (p < 0.01, Cochran-Armitage trend test).

For each antimicrobial class, we categorised the consumption based on different DDD cut-offs. The specific DDD cut-offs used for each antimicrobial drug class illustrate the average estimated number of DDD for one standard treatment for the most common infections.

**Table 2:** Association between antimicrobial prescription in the 3 years before HIV diagnosis and risk of HIV diagnosis, Denmark, 1998–2016 (n = 38,976)

	DDD/1 PY	First year before cases’ HIV diagnosis					Second year before cases’ HIV diagnosis					Third year before cases’ HIV diagnosis				
		HIV n = 2,784		Controls n = 36,192		OR (95% CI)	HIV n = 2,784		Controls n = 36,192		OR (95% CI)	HIV n = 2,784		Controls n = 36,192		OR (95% CI)
		n	%	n	%		n	%	n	%		n	%	n	%	
Beta-lactams	0	1,847	66.3	30,186	83.4	Ref (1)	2,029	72.9	30,137	83.3	Ref (1)	2,094	75.2	30,078	83.1	Ref (1)
	> 0–10	454	16.3	3,386	9.4	2.21 (1.98–2.46)	384	13.8	3,526	9.7	1.63 (1.45–1.83)	384	13.8	3,545	9.8	1.56 (1.39–1.75)
	> 10–20	316	11.4	1,963	5.4	2.64 (2.33–3.01)	252	9.1	1,888	5.2	2.00 (1.74–2.31)	219	7.9	1,939	5.4	1.63 (1.41–1.89)
	> 20	167	6.0	657	1.8	4.23 (3.54–5.05)	119	4.3	641	1.8	2.77 (2.27–3.39)	87	3.1	630	1.7	2.00 (1.59–2.52)
Macrolides	0	2,224	79.9	34,197	94.5	Ref (1)	2,407	86.5	34,136	94.3	Ref (1)	2,451	88.0	34,081	94.2	Ref (1)
	> 0–10	393	14.1	1,593	4.4	3.80 (3.38–4.28)	294	10.6	1,623	4.5	2.57 (2.25–2.93)	264	9.5	1,686	4.7	2.19 (1.91–2.50)
	> 10	167	6.0	402	1.1	6.45 (5.35–7.78)	83	3.0	433	1.2	2.73 (2.15–3.46)	69	2.5	425	1.2	2.26 (1.75–2.92)
Acyclovir	0	2,564	92.1	35,832	99.0	Ref (1)	2,656	95.4	35,834	99.0	Ref (1)	2,682	96.3	35,852	99.1	Ref (1)
	> 0–2	54	1.9	171	0.5	6.37(4.60–8.83)	36	1.3	125	0.4	3.98 (2.74–5.80)	30	1.1	111	0.3	3.68 (2.45–5.51)
	> 2	166	6.0	189	0.5	9.64 (7.88–11.79)	92	3.3	233	0.6	5.42 (4.24–6.94)	72	2.6	229	0.6	4.31 (3.29–5.64)
Azoles	0	2,579	92.6	35,578	98.3	Ref (1)	2,681	96.3	35,556	98.2	Ref (1)	2,707	97.2	35,561	98.3	Ref (1)
	> 0–3	78	2.8	410	1.1	2.79 (2.17–3.60)	49	1.8	426	1.2	1.57 (1.16–2.13)	36	1.3	412	1.1	1.16 (0.82–1.64)
	> 3	127	4.6	204	0.6	8.74 (6.97–10.96)	54	1.9	210	0.6	3.42 (2.53–4.63)	41	1.5	219	0.6	2.48 (1.77–3.47)
Quinolones	0	2,650	95.2	35,867	99.1	Ref (1)	2,704	97.1	35,871	99.1	Ref (1)	2,716	97.6	35,872	99.1	Ref (1)
	> 0–7	66	2.4	167	0.5	5.38 (4.04–7.18)	51	1.8	176	0.5	3.85 (2.81–5.28)	44	1.6	190	0.5	3.05 (2.20–4.25)
	> 7	68	2.4	158	0.4	5.92 (4.43–7.90)	29	1.0	145	0.4	2.67 (1.78–3.98)	24	0.9	130	0.4	2.47 (1.59–3.83)
Nystatin	0	2,690	96.6	36,118	99.8	Ref (1)	2,755	99.0	36,139	99.9	Ref (1)	2,773	99.6	36,141	99.9	Ref (1)
	> 0–4	39	1.4	41	0.1	13.44 (8.59–21.04)	14	0.5	29	0.1	6.47 (3.39–12.37)	6	0.2	30	0.1	2.62 (1.08–6.31)
	> 4	55	2.0	33	0.1	23.45 (15.08–36.46)	15	0.5	24	0.1	8.13 (4.26–15.49)	5	0.2	21	0.1	3.13 (1.17–8.36)
Doxycycline	0	2,719	97.7	36,057	99.6	Ref (1)	2,743	98.5	36,055	99.6	Ref (1)	2,746	98.6	36,052	99.6	Ref (1)
	> 0–20	41	1.5	101	0.3	5.37 (3.77–7.74)	23	0.8	97	0.3	3.12 (1.98–4.92)	23	0.8	116	0.3	2.63 (1.68–4.14)
	> 20	24	0.9	34	0.1	9.27 (5.49–15.63)	18	0.7	40	0.1	5.89 (3.38–10.28)	15	0.5	24	0.1	8.22 (4.31–15.67)
UTI drugs	0	2,660	95.6	35,350	97.7	Ref (1)	2,695	96.8	35,433	97.9	Ref (1)	2,695	96.8	35,422	97.9	Ref (1)
	> 0–5	33	1.2	283	0.8	1.59 (1.10–2.29)	38	1.4	296	0.8	1.73 (1.22–2.44)	34	1.2	321	0.9	1.42 (0.99–2.04)
	> 5	91	3.3	559	1.5	2.25 (1.79–2.84)	51	1.8	463	1.3	1.48 (1.10–1.99)	55	2.0	449	1.2	1.65 (1.23–2.20)

CI: confidence interval; DDD/1 PY: defined daily dose per person-year rate; HIV: human immunodeficiency virus; OR: unadjusted odds ratio; Ref: reference value; UTI: urinary tract infection.

We found a statistically significant association between the level of prescription of any of the analysed antimicrobial drugs and the risk of subsequent HIV diagnosis, which increased with a higher level of prescription and a shorter time to HIV diagnosis (Table 2).

**Figure 3:**
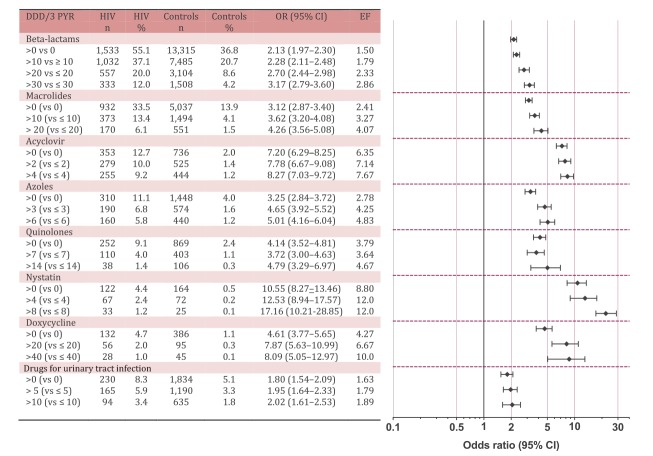
Association between different antimicrobial prescriptions in the 3 years before an HIV diagnosis and the risk of being subsequently diagnosed with HIV infection, Denmark, 1998–2016 (n = 38,976)

CI: confidence interval; DDD/3PYR: defined daily dose per person in the 3-year study period rate; EF: effectiveness factor; HIV: human immunodeficiency virus; OR: odds ratio.

In order to use estimates that could better translate into clinical practice, we analysed the exposure (i.e. consumption level) during the entire 3-year period as a binary exposure. The different DDD/PY cut-offs were based on the average estimated number of DDD for one or more treatments for the most commonly treated infections; the following results therefore illustrate the risk of a subsequent HIV diagnosis that was associated with the redemption of at least one treatment as compared with no treatment: beta-lactams (OR = 2.13; 95% CI: 1.97–2.30), macrolides (OR = 3.12; 95% CI: 2.87–3.40), acyclovir (OR = 7.20; 95% CI: 6.29–8.25), azoles (OR = 3.25; 95% CI: 2.84–3.72), quinolones (OR = 4.14; 95% CI: 3.52–4.81), nystatin (OR = 10.55; 95% CI: 8.27–13.46), doxycycline (OR = 4.61; 95% CI: 3.77–5.65) and UTI drugs (OR = 1.80; 95% CI: 1.54–2.09) (Figure 3). We found an overall strong and linear association between the magnitude of drug prescription and the risk of subsequent HIV diagnosis for all antimicrobial drugs with the exception of antibiotics used for UTI. For UTI drugs, even a higher cut-off (> 10 vs ≤ 10 DDD) had a low OR (2.02; 95% CI: 1.61–2.53). The analysis of the effectiveness factor showed a statistically significant association between targeted screening and subsequent HIV diagnosis, confirming how effective the different interventions would be compared with testing at random the whole population. The same analysis including only the second and third year before the HIV diagnosis is shown in Supplementary Table S2.

Percentages refer to the totals of all cases/all controls in this study.

### Sensitivity analyses

We performed several sensitivity analyses, i.e. stratifying results according to sex, age, mode of infection and HIV subgroups, for the data with statistically significant interactions and/or clinical interest.

**Figure 4:**
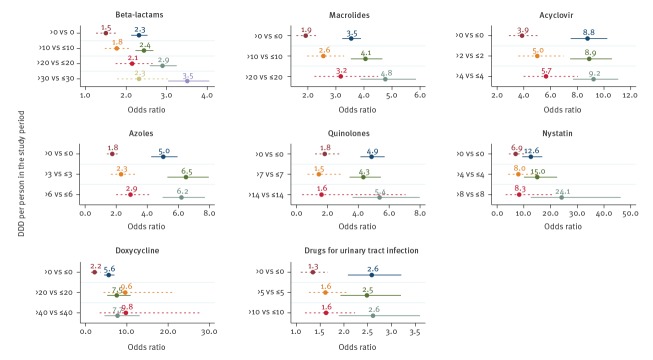
Association between antimicrobial prescription in the 3 years before HIV diagnosis and the risk of HIV diagnosis, stratified by sex, Denmark, 1998–2016 (n = 38,976)

CI: confidence interval; DDD: defined daily dose; UTI: urinary tract infection.

Firstly, we observed a similar association for men and women, although for most of the drugs, the association was significantly higher for men. However, it needs to be noted that the number of women in this study was small (Figure 4 and Supplementary Table S3).

Dashed lines: women, solid lines: men.

Secondly, a similar association was found for beta-lactams, macrolides, nystatin, doxycycline and UTI drugs irrespective of age and sex, when stratifying for both these variables. In contrast, in association with prescription of acyclovir, a much stronger association was found for middle-aged men (40–59 years; > 2 DDD/3 PY: OR: 13.22; 95% CI: 10.20–17.13). Regarding the prescription of azoles, the association increased with increasing age for both men and women. For the prescription of quinolones, the association was strongest in younger men (Supplementary Tables S4 and S5).

Thirdly, a stronger association between the prescription of antimicrobials and subsequent HIV diagnosis was observed for men who have sex with men (MSM) than for heterosexuals. This association was most pronounced for macrolides, quinolones and doxycycline (Supplementary Tables S6 and S7).

Finally, a strong association was observed for all HIV subgroups. Nevertheless, for some antimicrobial drugs (macrolides, acyclovir and azoles) a more pronounced association was observed for cases presenting with a late diagnosis (LHIV and VLHIV) (Supplementary Table S8).

## Discussion

In this nationwide, population-based nested case–control study, including 2,784 cases and 36,192 controls, we identified significantly higher levels of antimicrobial drug prescription for cases than controls in the 3 years preceding a new HIV diagnosis.

For all antimicrobial drug classes analysed, we found a statistically significant association between drug prescription and the risk of a subsequent HIV diagnosis. This association paralleled the level of prescription (i.e. increase in DDD/PY) and the proximity to the HIV diagnosis. The association was independent of age or Danish origin (data not shown), but more pronounced among men and those presenting with late-stage HIV infection. Overall, the prescription of antimicrobial drugs during the 3-year period was high for both cases and controls (72.4% of cases vs 46.3% of controls), and beta-lactams and macrolides were the most frequently used antimicrobial classes. 

In a recent study [25], we established that HIV-infected individuals had frequent contacts with PHC in the years preceding the HIV diagnosis. Identifying increased prescription of one of these antimicrobial drugs should raise awareness among primary care physicians of a potential occult HIV infection. Older age and heterosexual HIV-transmission were associated with late HIV diagnosis, probably because the clinical suspicion of occult HIV infection in these situations is lower. Proactive HIV screening based on a surrogate marker such as antibiotic prescription could therefore be useful.

Although, we had no information on the disease indication for the prescriptions in this study, we presume that the use of antimicrobial drugs may in many cases represent a proxy for missed HIV indicator conditions such as community-acquired pneumonia (CAP), STI, herpes simplex infections or candidiasis. Among MSM, we observed a stronger association between macrolides, quinolones and doxycycline use and subsequent HIV diagnosis compared with other antimicrobials, and in the case of quinolones, particularly among younger MSM. Although the controls were not matched for sexual preference, this could indicate that the consumption in these cases was driven by STI, which in itself should prompt an HIV test. In addition, we assume that acyclovir was used for herpes simplex and zoster virus infections and azoles mainly for *Candida* infections and these antimicrobials could therefore serve as proxies for indicator conditions. Different clinical indications are plausible for beta-lactam and macrolides use; however, we suspect that a large proportion may have been given for CAP.

Our study provides additional evidence concerning missed opportunities for earlier HIV diagnosis, regarding both the identification of indicator conditions and the identification of behavioural aspects and risk factors for HIV infection. These findings support that a new targeted strategy is needed to find people with undiagnosed HIV infection in the general population in order to improve timely diagnosis and avoid new onward transmissions.

A recent analysis in Denmark has shown that a large percentage of people newly diagnosed with HIV has visited PHC or even hospitals 2 years before the diagnosis without being tested for HIV, although they presented with some clear indicator conditions [25,30]. Therefore, the strategy suggested in our analysis should be complementary to the mandatory HIV testing in people with indicator conditions.

The Centers for Disease Control and Prevention (CDC) in the US recommend universal HIV screening at least once during adulthood when in contact with any healthcare setting. However, this practice has so far not been widely implemented [31-33]. Furthermore, the individual HIV risk may vary during the lifetime if an individual develops new risk practices and one random HIV test may not capture the patient when at risk. Our data indicate that prescription of some antimicrobial drugs, and in particular repeated use over a short time interval, could be considered a marker of increased risk of occult HIV infection and act as a reminder in both primary and secondary healthcare to consider HIV testing; this would make the risk assessment a more dynamic process throughout the lifespan of sexually active adults.

In most European countries with a low HIV prevalence, targeted HIV testing is recommended based on identifying indicator conditions and risk groups. Nevertheless, many missed opportunities for HIV testing occur in these situations despite the existing recommendation, as highlighted in previous studies [21,22,34]. Based on our results, it seems reasonable to perform an HIV test after prescription of acyclovir, azoles, nystatin, doxycline, quinolones and macrolides. Even for women, whose risk was lower, the results were still statistically significant, although it has to be noted that the number of women in this study was small. Furthermore, recurrent beta-lactam use, where we suggest a threshold above two prescriptions in a 1–2-year period, may also be used as an indicator to perform an HIV test. The antimicrobial consumption in these situations was associated with a high risk of HIV with an OR > 2, both in the analysis of the cumulative data for all the 3 years before HIV diagnosis and in the analysis including only the second and third year before diagnosis. Analysis of the effectiveness factor of these targeted prescriptions confirmed how effective the different interventions would be compared with testing at random. Given an HIV prevalence of 0.1% in the general Danish population, HIV prevalence in these subgroups could be estimated at above 0.2% (above 0.4% in the case of quinolone, doxycycline, acyclovir and nystatin consumption), which is regarded as a cost-effective strategy [13-15]. The prescription of these antimicrobials is an easily recognisable parameter, especially when using electronic health records. This might help the physician identify individuals at risk, and automatic reminders could easily be introduced into the system. However, in countries without electronic health records, these data may not be so easily available. Further studies are needed to confirm if this approach is cost-effective.

The main strengths of our study include its design with nesting in a well-established nationwide population-based HIV cohort and access to a well-matched control group from the population. We had full access to Danish registries of high quality, allowing us to look 3 years back in time from the established HIV diagnosis. With the DNPR, we had access to valid, nationwide, individual-level data on all dispensed antimicrobial prescriptions since 1995. Redemption data have proven to be a good proxy for consumption as primary non-adherence to antimicrobial drugs in Denmark is rare (6.5%) and antimicrobial drugs are available only on prescription from Danish physicians [35]. Furthermore, the DNPR includes information on redeemed rather than on issued prescriptions, which constitutes a more accurate surrogate measure for actual antimicrobial consumption, as around 10% of issued prescriptions in Denmark are not subsequently redeemed in the pharmacies [29]. Of note, our study includes vulnerable populations such as people who inject drugs and migrants. We show that even though these populations probably have risk factors that should have triggered an HIV test, they do attend primary healthcare and they could be captured by their antimicrobial consumption. To the best of our knowledge, this is the first study to analyse this.

Our study has some limitations. Some antimicrobial drugs (i.e. beta-lactams, macrolides, quinolones and doxycycline) may have several indications (e.g. CAP, skin infections, UTI, STI). Data on the disease indication for the drug prescriptions or presence of indicator conditions is not available in the DNPR. As a result, the DDD cut-offs for each antimicrobial class, were chosen based on the average estimated number of DDDs for one treatment for the most common conditions, may not be entirely correct. However, the same sources of data were used for both cases and controls, minimising differential misclassification. Unfortunately, we had no information regarding sexual orientation among controls and hence no knowledge of the percentage of MSM among controls. Another potential shortcoming is that we included only individuals living in Denmark 3 years or more before HIV diagnosis in the matched case. Excluding individuals who emigrated or immigrated in this period should, however, not affect our results, as we did not observe significant differences in antimicrobial consumption between non-Danish and Danish cases.

## Conclusion

This case–control study nested in the nationwide DHCS identified a significantly higher consumption of many antimicrobial drugs along the 3 years preceding a new HIV diagnosis. The antimicrobial drug consumption was associated with a subsequent risk of HIV diagnosis. In many cases, the consumption may represent a proxy for an HIV indicator condition and thus would clearly identify a missed opportunity for timely diagnosis of HIV infection and ART initiation. We suggest that antimicrobial drug prescription, a parameter easily monitored in electronic medical records, could be considered as part of the individual risk assessment and trigger proactive HIV testing in PHC. Further studies are needed to confirm the relevance and cost-benefit of such an approach.

## Supplementary Data

366000 Supplement
