# Supplementary material for: Prescription of antimicrobials in primary health care as a marker to identify people living with undiagnosed HIV infection, Denmark, 1998 to 2016
Source: Euro Surveill. 2019 Oct 10;24(41):1900225. doi: 10.2807/1560-7917.ES.2019.24.41.1900225 (PMC6794988; doi:10.2807/1560-7917.ES.2019.24.41.1900225)
Supplement: Supplement [file 19-00225_MARTIN-IGUACEL_SupplementaryTables.pdf]

Presciption of antimicrobials in primary health care as a marker of occult HIV infection. Supplementary material.

This supplementary material is hosted by Eurosurveillance as supporting information alongside the article *Prescription of antimicrobials in primary health care as a marker to identify people living with undiagnosed HIV infection, Denmark, 1998 to 2016,* on behalf of the authors who remain responsible for the accuracy and appropriateness of the content. The same standards for ethics, copyright, attributions and permissions as for the article apply. Supplements are not edited by *Eurosurveillance* and the journal is not responsible for the maintenance of any links or email addresses provided therein.

Supplementary table S1

All classes of antimicrobial drugs were estimated. However, we only included those drug classes that had been used by more than 1% of the HIV patients in the study period for further analyses.

The following antimicrobials were included:

**Name**	**Fifth ATC classification level**
Beta-lactams:	
Phenoxymethylpenicillin	J01CE02
Ampicillin	J01CA01
Pivampicillin	J01CA02
Amoxicillin	J01CA04
Amoxicillin/clavulanic acid	J01CR02
Macrolides:	
Erythromycin	J01FA01
Clarithromycin	J01FA09
Azithromycin	J01FA10
Roxithromycin	J01FA06
Rovamycin	J01FA02
Fluoroquinolones:	
Ciprofloxacin	J01MA02
Moxifloxacin	J01MA14
Ofloxacin	J01MA01
Norfloxacin	J01MA06
Fleroxacin	J01MA08
Grepafloxacin	J01MA11
Antivirals	
Acyclovir	J05AB01
Valacyclovir	J05AB11
Azoles:	

Ketoconazole	J02AB02
Fluconazole	J02AC01
Itraconazole	J02AC02
Voriconazole	J02AC03
Posaconazole	J02AC04
Nystatin	
Nystatin	A07AA02
Tetracycline:	
Doxycycline	J01AA02
Tetracycline	J01AA07
Drugs specific used for urinary tract infections	
Pivmecillinam	J01CA08
Trimethoprim	J01EA01
Sulfamethizole	J01EB02
Nitrofurantoin	J01XE01
Methenamine	J01XX05

The following antibiotic classes were not included because of limited use (<1%):

**Name**	**Fifth ATC classification level**
Oral cephalosporines:	
Cefalexin	J01DB01
Cefuroxim	J01DC02
Lincosamides:	
Clindamycin	J01FF01
Imidazol derivates	
Metronidazole	P01AB01
Combination of sulphonamides and trimethoprim:	
Trimethoprim/sulfamethoxazole	J01EE01
Beta-lactamase resistant Penicillins:	
Dicloxacillin	J01CF01
Fusidin	J01XC01
Amphenicols:	
Chloranphenicol	J01BA01
Antiviral for cytomegalovirus Infections:	
Valganciclovir	J05AB14
Oxazolidinone derivate:	
Linezolid	J01XX08

For each antimicrobial drug class, we categorized the consumption based on different DDD cut-offs. These cut-offs were chosen based on clinical criteria as the average estimated number of DDDs for one standard treatment for the most common infections: 1) beta-lactams: 0, >0-10, >10-20, >20 DDD/PY (10 DDD/PY equals 7-10 days treatment for uncomplicated community-acquired pneumonia (CAP)); 2) macrolides: 0, >0-10, >10 DDD/PY (10 DDD/PY equals a 5-10 days treatment for uncomplicated CAP); 3) antivirals: 0, >0-2, >2 DDD/PY (2 DDD/PY equals a treatment for herpes simplex labialis); 4) azoles: 0, >0-3, >3 DDD/PY (3 DDD/PY equals one week treatment for Candida infection); 5) quinolones: 0, >0-7, >7 DDD/PY (7 DDD/PY equals one week treatment); 6) tetracycline: 0, >0-20, >20 DDD/PY (20 DDD/PY equals one week treatment); 7) Nystatin: 0, >0-4, >4 DDD/PY (4 DDD/PY equals 14 days treatment); 8) UTI drugs: 0, >0-5, >5 DDD/PY (5 DDD/PY equals 5 days treatment for uncomplicated

Supplementary table S2. Association between different antimicrobial prescriptions in the 2nd and 3rd years before HIV diagnosis and the risk of being subsequently diagnosed with HIV infection.

HIV vs· Controls in the 2nd and 3rd years before HIV dx				
	DDD/2PY	HIV (N=2784)	Controls (N=36192)	OR (95% CI)
**Beta-lactams**				
	0	1647 (59·2)	25926 (71·6)	Ref (1)
	>0 vs 0	1137 (40·8)	10266 (28·4)	1·76 (1·63-1·91)
	>10 vs ≤ 10	655 (23·5)	5113 (14·1)	1·89 (1·72-2·07)
	>20 vs ≤ 20	286 (10·3)	1717 (4·7)	2·32 (2·03-2·65)
	>30 vs ≤ 30	140 (5·0)	772 (2·1)	2·44 (2·03-2·93)
**Macrolides**				
	0	2180 (78·3)	32484 (89·8)	Ref (1)
	>0 (vs 0)	604 (21·7)	3708 (10·3)	2·43 (2·20-2·67)
	>10 (vs ≤10)	177 (6·4)	968 (2·7)	2·47 (2·10-2·92)
	> 20 (vs ≤20)	67 (2·4)	327 (0·9)	2·71 (2·08-3·54)
**Acyclovir**				
	0	2586 (92·9)	35643 (98·5)	Ref (1)
	>0 (vs 0)	198 (7·1)	549 (1·5)	5·13 (4·33-6·09)
	>2 (vs ≤2)	151 (5·4)	384 (1·1)	5·46 (4·49-6·64)
	>4 (vs ≤ 4)	137 (4·9)	309 (0·9)	6·16 (5·00-7·58)
**Azoles**				
	0	2629 (94·4)	35121 (97·0)	Ref (1)
	>0 (vs 0)	155 (5·6)	1071 (3·0)	2·00 (1·67-2·39)
	>3 (vs ≤3)	84 (3·0)	414 (1·1)	2·71 (2·13-3·44)
	>6 (vs ≤ 6)	70 (2·5)	320 (0·9)	2·91 (2·23-3·38)
**Quinolones**				
	0	2642 (94·9)	35585 (98·3)	Ref (1)
	>0 (vs 0)	142 (5·1)	607 (1·7)	3·19 (2·64-3·85)
	>7 (vs ≤7)	53 (1·9)	270 (0·8)	2·61 (1·93-3·52)
	>14 (vs ≤14)	14 (0·5)	71 (0·2)	2·60 (1·46-4·63)
**Nystatin**				
	0	2746 (98·6)	36096 (99·7)	Ref (1)
	>0 (vs 0)	38 (1·4)	96 (0·3)	5·25 (3·59-7·67)
	>4 (vs ≤4)	19 (0·7)	42 (0·1)	5·94 (3·45-10·24)
	>8 (vs ≤8)	8 (0·3)	16 (0·04)	6·50 (2·78-15·19)
**Doxycycline**				
	0	2711 (97·4)	35925 (99·3)	Ref (1)
	>0 (vs 0)	73 (2·6)	267 (0·7)	3·62 (2·79-4·71)
	>20 (vs ≤20)	33 (1·2)	65 (0·2)	6·69 (4·39-10·20)
	>40 (vs ≤40)	21 (0·8)	32 (0·1)	8·53 (4·92-14·79)
**UTI drugs**				

	0	2554 (91·7)	34358 (94·9)	Ref (1)
	>0 (vs 0)	230 (8·3)	1834 (5·1)	1·63 (1·36-1·95)
	> 5 (vs ≤5)	165 (5·9)	1190 (3·3)	1·65 (1·32-2·06)
	>10 (vs ≤10)	94 (3·4)	635 (1·8)	1·71 (1·28-2·30)

Abbreviations: CI, confidence interval; DDD/2PY, defined daily dose per person in the 2nd and 3rd years before HIV diagnosis; OR, unadjusted odds ratio; UTI, urinary tract infection.

Supplementary table S3. Association between antimicrobial prescription in the 3 years before HIV diagnosis and the risk of being diagnosed with HIV infection stratified by sex (HIV vs. matched controls).

		Last year before HIV diagnosis			2nd year before HIV diagnosis			3rd year before HIV diagnosis		
	**DDD/1PYR**	**Men OR (95% CI)**	**Women OR (95% CI)**	**p-value[1]**	**Men OR (95% CI)**	**Women OR (95% CI)**	**p-value[1]**	**Men OR (95% CI)**	**Women OR (95% CI)**	**p-value[1]**
**Beta-lactams**	0	Ref (1)	Ref (1)		Ref (1)	Ref (1)		Ref (1)	Ref (1)	
	>0-10	2·33 (2·06-2·63)	1·76 (1·38-2·25)	0·046	1·75 (1·54-1·99)	1·24 (0·95-1·60)	0·019	1·75 (1·55-1·99)	0·98 (0·74-1·29)	<0·001
	>10-20	2·82 (2·45-3·26)	2·05 (1·54-2·73)	0·050	2·02 (1·72-2·37)	1·93 (1·45-2·57)	0·795	1·66 (1·40-1·96)	1·51 (1·11-2·05)	0·600
	>20	4·82 (3·93-5·91)	2·83 (1·96-4·08)	0·013	2·98 (2·36-3·75)	2·22 (1·47-3·35)	0·225	2·23 (1·71-2·90)	1·43 (0·89-2·30)	0·111
**Macrolides**	0	Ref (1)	Ref (1)		Ref (1)	Ref (1)		Ref (1)	Ref (1)	
	>0-10	4·39 (3·85-5·01)	2·21 (1·67-2·92)	<0·001	3·00 (2·60-3·47)	1·41 (1·03-1·94)	<0·001	2·50 (2·15-2·92)	1·35 (0·98-1·84)	<0·001
	>10	7·43 (5·99-9·21)	4·17 (2·83-6·16)	0·011	2·77 (2·09-3·68)	2·54 (1·62-3·98)	0·745	2·34 (1·73-3·18)	2·02 (1·25-3·26)	0·606
**Acyclovir**	0	Ref (1)	Ref (1)		Ref (1)	Ref (1)		Ref (1)	Ref (1)	
	>0-2	9·31 (6·44-13·45)	1·75 (0·74-4·14)	<0·001	6·09 (3·91-9·48)	1·61 (0·73-3·54)	<0·001	5·01 (3·17-7·94)	1·56 (0·61-3·96)	0·028
	>2	11·30 (8·97-14·23)	5·79 (3·76-8·91)	<0·001	6·14 (4·65-8·11)	3·53 (2·05-6·08)	0·075	4·13 (5·63)	4·96 (2·84-8·69)	0·571
**Azoles**	0	Ref (1)	Ref (1)		Ref (1)	Ref (1)		Ref (1)	Ref (1)	
	>0-3	5·21 (3·58-7·59)	1·81 (1·28-2·55)	<0·001	2·70 (1·60-4·55)	1·24 (0·85-1·80)	0·018	1·57 (0·86-2·87)	1·02 (0·67-1·55)	0·248
	>3	12·30 (9·36-16·18)	3·92 (2·50-6·15)	<0·001	4·81 (3·37-6·86)	1·67 (0·91-3·07)	<0·001	2·68 (1·78-4·04)	2·12 (1·17-3·38)	0·519
**Quinolones**	0	Ref (1)	Ref (1)		Ref (1)	Ref (1)		Ref (1)	Ref (1)	
	>0-7	6·27 (4·59-8·59)	2·63 (1·23-5·65)	0·039	4·62 (3·27-6·52)	1·88 (0·85-4·18)	0·043	3·53 (2·48-5·04)	1·48 (0·58-3·75)	0·086
	>7	7·32 (5·38-9·96)	1·46 (0·52-4·12)	<0·001	2·74 (1·77-4·22)	2·30 (0·79-6·69)	0·767	2·95 (1·87-4·66)	0·52 (0·07-3·85)	0·098
**Nystatin**	0	Ref (1)	Ref (1)		Ref (1)	Ref (1)		Ref (1)	Ref (1)	
	>0-4	17·39 (10·16-29·78)	7·06 (2·97-16·76)	0·083	5·97 (2·70-13·23)	7·64 (2·48-23·48)	0·727	2·18 (0·64-7·48)	3·25 (0·92-11·52)	0·659
	>4	28·90 (16·79-49·75)	14·52 (6·62-31·87)	0·158	9·18 (4·38-19·21)	5·57 (1·44-21·55)	0·526	4·00 (1·30-12·27)	1·64 (0·20-13·50)	0·465
**Doxycycline**	0	Ref (1)	Ref (1)		Ref (1)	Ref (1)		Ref (1)	Ref (1)	
	>0-20	6·65 (4·39-10·07)	2·77 (1·22-6·29)	0·062	3·88 (2·38-6·32)	1·02 (0·24-4·32)	0·086	3·55 (2·13-5·91)	1·17 (0·42-3·27)	0·058
	>20	10·56 (5·86-19·03)	5·78 (1·78-18·76)	0·369	4·73 (2·51-8·92)	16·26 (4·36-60·53)	0·098	8·75 (4·45-17·22)	4·36 (0·45-41·94)	0·563
**UTI drugs**	0	Ref (1)	Ref (1)		Ref (1)	Ref (1)		Ref (1)	Ref (1)	
	>0-5	3·00 (1·64-5·49)	1·19 (0·75-1·90)	0·018	2·97 (1·58-5·55)	1·43 (0·95-2·17)	0·058	1·78 (0·89-3·58)	1·31 (0·86-2·00)	0·457
	>5	2·97 (2·14-4·13)	1·76 (1·27-2·45)	0·027	1·61 (1·01-2·28)	1·39 (0·95-2·04)	0·634	2·07 (1·34-3·21)	1·41 (0·96-2·07)	0·191

Abbreviations: CI, confidence interval; DDD/1PYR, defined daily dose per person-year rate; OR, unadjusted odds ratio; UTI, urinary tract infection.

[1] p-value (wald test) in the different strata testing if the association between antimicrobial drug prescription and subsequent HIV diagnosis is statistically significantly different in women and men.

The overall likelihood ratio test (LRT) for sex differences in the 3 years period was only significant (<0·01) for beta-lactams, macrolides, azoles.

Supplementary table S4. Association between antimicrobial prescription in the 3 years before HIV diagnosis and the risk of being diagnosed with HIV infection stratified by age (HIV vs. matched controls).

		18-39 years			40-59 years				≥ 60 years			
	DDD/3PY	HIV N= 1478	Control N=19231	OR (95% CI)	HIV N= 1108	Control N=14393	OR (95% CI)	p-value[1]	HIV N= 198	Control N=2568	OR (95% CI)	p-value[1]
**Beta-lactams**												
	0	701 (47·4)	12359 (64·3)	Ref (1)	465 (42·0)	8980 (62·4)	Ref (1)		85 (42·9)	1538 (59·9)	Ref (1)	
	>0-10	260 (17·6)	3079 (16·0)	1·52 (1·31-1·76)	212 (19·1)	2356 (16·4)	1·74 (1·47-2·06)	0·24	29 (14·7)	395 (15·4)	1·34 (0·87-2·06)	0·59
	>10-20	242 (16·4)	2222 (11·6)	1·96 (1·68-2·29)	197 (17·8)	1823 (12·7)	2·08 (1·75-2·48)	0·64	36 (18·2)	336 (13·1)	1·99 (1·32-3·01)	0·65
	>20	275 (18·6)	1571 (8·2)	3·20 (2·75-3·72)	234 (21·1)	1234 (8·6)	3·67 (3·10-4·34)	0·24	48 (24·2)	299 (11·6)	2·92 (2·00-4·25)	0·61
**Macrolides**												
	0	995 (67·3)	16531 (86·0)	Ref (1)	724 (65·3)	12438 (86·4)	Ref (1)		133 (67·2)	2186 (85·1)	Ref (1)	
	>0-10	305 (20·6)	1967 (10·2)	2·58 (2·55-2·96)	221 (20·0)	1348 (9·4)	2·81 (2·39-3·30)	0·43	33 (16·7)	228 (8·9)	2·37 (1·58-3·55)	0·69
	>10	178 (12·0)	733 (3·8)	4·05 (3·40-4·83)	163 (14·7)	607 (4·2)	4·67 (3·86-5·65)	0·28	32 (16·2)	154 (6·0)	3·44 (2·26-5·25)	0·49
**Acyclovir**												
	0	1316 (89·0)	18807 (97·8)	Ref (1)	943 (85·1)	14139 (98·2)	Ref (1)		172 (86·9)	2510 (97·4)	Ref (1)	
	>0-2	36 (2·4)	138 (0·7)	3·80 (2·62-5·51)	29 (2·6)	67 (0·5)	6·70 (4·28-10·47)	0·06	9 (4·6)	6 (0·2)	20·81 (7·38-58·64)	<0·01
	>2	126 (8·5)	286 (1·5)	6·56 (5·25-8·20)	136 (12·3)	187 (1·3)	11·22 (8·86-14·21)	<0·01	17 (8·6)	52 (2·0)	4·70 (2·65-8·33)	0·29
**Azoles**												
	0	1357 (91·8)	18331 (95·3)	Ref (1)	951 (85·8)	13909 (96·6)	Ref (1)		166 (83·8)	2504 (97·5)	Ref (1)	
	>0-3	66 (4·5)	591 (3·1)	1·58 (1·21-2·07)	46 (4·2)	257 (1·8)	2·90 (2·08-4·03)	<0·01	8 (4·0)	26 (1·0)	4·51 (2·00-10·19)	0·02
	>3	55 (3·7)	309 (1·6)	2·48 (1·84-3·33)	111 (10·0)	227 (1·6)	7·43 (5·84-9·45)	<0·01	24 (12·1)	38 (1·5)	9·77 (5·65-16·89)	<0·01
**Quinolones**												
	0	1354 (91·6)	18888 (98·2)	Ref (1)	1003 (90·5)	14002 (97·3)	Ref (1)		175 (88·4)	2433 (94·7)	Ref (1)	
	>0-7	79 (5·4)	220 (1·1)	5·11 (3·91-6·67)	57 (5·1)	192 (1·3)	4·19 (3·09-5·67)	0·33	6 (3·03)	54 (2·1)	1·53 (0·66-3·64)	0·01
	>7	45 (3·0)	123 (0·6)	5·21 (3·67-7·41)	48 (4·3)	199 (1·4)	3·42 (2·47-4·72)	0·09	17 (17·4)	81 (3·2)	2·94 (1·70-5·09)	0·09
**Nystatin**												
	0	1433 (97·0)	19158 (99·6)	Ref (1)	1050 (94·8)	14321 (99·5)	Ref (1)		179 (90·4)	2549 (99·3)	Ref (1)	
	>0-4	22 (1·5)	49 (0·3)	6·24 (3·74-10·41)	26 (2·4)	34 (0·2)	11·14 (6·58-18·86)	0·12	7 (3·5)	9 (0·4)	12·07 (4·30-33·88)	0·26
	>4	23 (1·6)	24 (0·1)	12·91 (7·28-22·90)	32 (2·9)	38 (0·3)	11·78 (7·30-19·0)	0·81	12 (6·1)	10 (0·4)	19·40 (7·84-47·99)	0·46
**Doxycycline**												
	0	1413 (95·6)	19028 (98·9)	Ref (1)	1051 (94·9)	14241 (98·9)	Ref (1)		188 (95·0)	2537 (98·8)	Ref (1)	
	>0-20	36 (2·4)	156 (0·8)	3·10 (2·15-4·47)	35 (3·2)	116 (0·8)	4·02 (2·74-5·91)	0·33	5 (2·5)	19 (0·7)	3·57 (1·33-9·59)	0·79
	>20	29 (2·0)	47 (0·2)	8·33 (5·22-13·30)	22 (2·0)	36 (0·3)	8·29 (4·84-14·20)	0·99	5 (2·5)	12 (0·5)	5·81 (2·01-16·78)	0·54
**UTI drugs**												
	0	1366 (92·4)	18202 (94·7)	Ref (1)	1021 (92·2)	13798 (95·9)	Ref (1)		167 (84·3)	2358 (91·8)	Ref (1)	
	>0-5	36 (2·4)	36 (2·4)	1·21 (0·85-1·72)	23 (2·1)	189 (1·3)	1·75 (1·12-2·74)	0·20	6 (3·0)	38 (1·5)	2·30 (0·95-5·57)	0·18
	>5	76 (5·1)	76 (5·1)	1·76 (1·35-2·28)	64 (5·8)	406 (2·8)	2·26 (1·70-2·99)	0·21	25 (12·6)	172 (6·7)	2·17 (1·34-3·46)	0·44

Abbreviations: CI, confidence interval; DDD/3PYR, defined daily dose per person in the three-years study period rate; OR, unadjusted odds ratio; UTI, urinary tract infection.

[1] p-value (wald test) in the different strata testing if the association between antimicrobial drug prescription and subsequent HIV diagnosis is statistically significantly different in the different age groups (comparing with the reference age group 18-39).

The overall LRT for age differences in antimicrobial drug consumption in the 3 years period was significant (<0·01) for azoles and acyclovir.

Supplementary table S5. Association between antimicrobial prescription in the 3 years before HIV diagnosis and the risk of being diagnosed with HIV infection stratified by sex and age (HIV vs. matched controls).

		**MEN**					**WOMEN**				
		**18-39**	**40-59**		**≥ 60**		**18-39**	**40-59**		**≥ 60**	
	DDD/3PY	OR (95% CI)	OR (95% CI)	p-value[1]	OR (95% CI)	p-value[1]	OR (95% CI)	OR (95% CI)	p-value[1]	OR (95% CI)	p-value[1]
**Beta-lactams**											
	0	Ref (1)	Ref (1)		Ref (1)		Ref (1)	Ref (1)		Ref (1)	
	>0-10	1·73 (1·46-2·04)	1·79 (1·49-2·15)	0·78	1·47 (0·93-2·31)	0·51	0·98 (0·72-1·34)	1·43 (0·90-2·28)	0·19	0·60 (0·13-2·74)	0·53
	>10-20	2·27 (1·91-2·70)	2·11 (1·75-2·55)	0·56	2·29 (1·49-3·51)	0·98	1·19 (0·85-1·65)	1·91 (1·21-3·01)	0·10	0·36 (0·04-2·85)	0·26
	>20	3·87 (3·24-4·63)	3·68 (3·06-4·43)	0·70	3·05 (2·04-4·56)	0·29	1·91 (1·44-2·53)	3·52 (2·33-5·32)	0·02	2·06 (0·72-5·86)	0·89
**Macrolides**											
	0	Ref (1)	Ref (1)		Ref (1)		Ref (1)	Ref (1)		Ref (1)	
	>0-10	3·12 (2·68-3·65)	3·11 (2·62-3·70)	0·98	2·20 (1·41-3·41)	0·14	1·41 (1·05-1·90)	1·56 (1·00-2·43)	0·73	3·69 (1·28-10·61)	0·09
	>10	4·87 (3·95-5·99)	5·08 (4·11-6·27)	0·77	3·58 (2·27-5·65)	0·23	2·55 (1·82-3·55)	3·14 (2·01-4·90)	0·46	3·01 (0·97-9·29)	0·78
**Acyclovir**											
	0	Ref (1)	Ref (1)		Ref (1)		Ref (1)	Ref (1)		Ref (1)	
	>0-2	5·73 (3·73-8·80)	8·97 (5·43-14·82)	0·18	21·18 (7·11-66·71)	0·03	1·40 (0·60-3·26)	2·44 (0·83-7·22)	0·43	11·97 (0·75-191·77)	0·15
	>2	7·08 (5·42-9·25)	13·22 (10·20-17·13)	<0·01	5·42 (3·02-9·75)	0·42	5·51 (3·30-8·20)	4·93 (2·66-9·14)	0·77	-	-
**Azoles**											
	0	Ref (1)	Ref (1)		Ref (1)		Ref (1)	Ref (1)		Ref (1)	
	>0-3	2·64 (1·72-4·03)	4,40 (2·96-6·53)	0·10	3·35 (1·36-8·22)	0·64	1·26 (0·91-1·73)	1·77 (1·07-2·91)	0·26	9·54 (1·94-46·83)	0·01
	>3	3·77 (2·56-5·55)	8·65 (6·49-11·53)	<0·01	10·21 (5·57-18·73)	<0·01	1·35 (0·76-2·41)	5·88 (3·50-9·87)	<0·01	33·57 (3·42-329·23)	<0·01
**Quinolones**											
	0	Ref (1)	Ref (1)		Ref (1)		Ref (1)	Ref (1)		Ref (1)	
	>0-7	6·51 (4·81-8·80)	4·75 (3·44-6·56)	0·16	1·79 (0·75-4·25)	<0·01	2·39 (1·30-4·37)	1·84 (0·72-4·73)	0·65	-	0·98
	>7	6·43 (4·34-9·53)	4·06 (2·90-5·68)	0·08	3·24 (1·83-5·74)	0·05	2·51 (1·10-5·72)	0·42 (0·06-3·05)	0·10	1·14 (0·14-9·28)	0·49
**Nystatin**											
	0	Ref (1)	Ref (1)		Ref (1)		Ref (1)	Ref (1)		Ref (1)	
	>0-4	8·17 (4·19-15·96)	11·12 (6·03-20·50)	0·51	13·24 (4·57-38·32)	0·45	4·39 (1·95-9·87)	11·05 (3·95-30·93)	0·17	-	0·98
	>4	15·73 (7·47-33·12)	13·56 (7·90-23·28)	0·75	26·11 (8·85-77·02)	0·45	9·68 (3·89-24·09)	6·97 (2·37-20·49)	0·65	7·26 (1·19-44·37)	0·78
**Doxycycline**											
	0	Ref (1)	Ref (1)		Ref (1)		Ref (1)	Ref (1)		Ref (1)	
	>0-20	5·17 (3·45-7·76)	4·41 (2·91-6·66)	0·60	3·93 (1·45-10·66)	0·62	0·56 (0·18-1·79)	2·40 (0·82-7·06)	0·07	-	0·98
	>20	7·52 (4·44-12·74)	8·54 (4·78-15·24)	0·75	5·95 (1·83-19·36)	0·72	12·94 (4·54-36·88)	7·04 (1·66-29·83)	0·50	4·76 (0·41-55·06)	0·46
**UTI drugs**											
	0	Ref (1)	Ref (1)		Ref (1)		Ref (1)	Ref (1)		Ref (1)	
	>0-5	2·65 (1·42-4·94)	3·47 (1·96-6·16)	0·53	1·52 (0·46-5·05)	0·42	0·90 (0·59-1·38)	0·84 (0·40-1·76)	0·88	6·06 (1·41-26·01)	0·01
	>5	3·19 (2·01-5·05)	2·59 (1·81-3·70)	0·46	1·75 (1·00-3·06)	0·10	1·39 (1·02-1·90)	1·78 (1·15-2·78)	0·37	4·68 (1·76-12·44)	0·02

Abbreviations: CI, confidence interval; DDD/3PYR, defined daily dose per person in the three-years study period rate; OR, unadjusted odds ratio; UTI, urinary tract infection.

[1] p-value (wald test) in the different strata testing if the association between antimicrobial drug prescription and subsequent HIV diagnosis is statistically significantly different the different age groups (comparing with the reference age group 18-39) stratified by sex.

The overall likelihood ratio test (LRT) for age differences in antimicrobial drug consumption in the 3 years period prior to HIV diagnosis was significant (LRT<0·01) only in men for the following antimicrobials acyclovir, azoles and nystatin. No significant differences were observed for women.

Supplementary table S6. Association between antimicrobial prescription in the 3 years before HIV diagnosis and the risk of being diagnosed with HIV infection stratified by mode of infection (HIV vs. matched controls).

		MSM	HTX		IDU		Unknown	
	DDD/3PYR	OR (95% CI)	OR (95% CI)	p-value[1]	OR (95% CI)	p-value[1]	OR (95% CI)	p-value[1]
**Beta-lactams**								
	0	Ref (1)	Ref (1)		Ref (1)		Ref (1)	
	>0-10	1·81 (1·56-2·11)	1·41 (1·18-1·69)	0·04	1·38 (0·90-2·10)	0·23	1·27 (0·82-1·95)	0·12
	>10-20	2·37 (2·03-2·77)	1·64 (1·36-1·98)	0·003	1·70 (1·08-2·67)	0·17	2·03 (1·37-3·01)	0·48
	>20	3·63 (3·10-4·27)	3·03 (2·56-3·60)	0·13	3·97 (2·69-5·86)	0·68	2·73 (1·83-4·07)	0·19
**Macrolides**								
	0	Ref (1)	Ref (1)		Ref (1)		Ref (1)	
	>0-10	3·63 (3·16-4·16)	1·89 (1·58-2·25)	<0·001	1·67 (1·10-2·54)	0·001	2·45 (1·66-3·60)	0·06
	>10	5·27 (4·41-6·30)	3·70 (3·05-4·50)	0·009	1·71 (0·89-3·28)	0·001	3·82 (2·44-5·97)	0·19
**Acyclovir**								
	0	Ref (1)	Ref (1)		Ref (1)		Ref (1)	
	>0-2	7·81 (5·23-11·67)	4·27 (2·76-6·59)	0·05	1·09 (0·25-4·62)	0·01	5·74 (2·38-13·81)	0·53
	>2	9·17 (7·34-11·45)	8·45 (6·54-10·91)	0·64	2·55 (1·22-5·34)	0·001	6·66 (4·05-10·96)	0·25
**Azoles**								
	0	Ref (1)	Ref (1)		Ref (1)		Ref (1)	
	>0-3	3·35 (2·32-4·84)	1·86 (1·43-2·43)	0·01	0·61 (0·24-1·55)	0·001	2·91 (1·36-6·23)	0·75
	>3	6·05 (4·62-7·91)	4·09 (3·15-5·29)	0·04	1·31 (0·46-3·68)	0·005	7·48 (4·41-12·68)	0·48
**Quinolones**								
	0	Ref (1)	Ref (1)		Ref (1)		Ref (1)	
	>0-7	6·87 (5·29-8·91)	2·76 (1·95-3·91)	<0·001	2·34 (0·97-5·66)	0·02	2·34 (1·04-5·26)	0·01
	>7	5·19 (3·85-7·01)	3·31 (2·27-4·80)	0·07	1·21 (0·28-5·20)	0·06	2·76 (1·38-5·55)	0·10
**Nystatin**								
	0	Ref (1)	Ref (1)		Ref (1)		Ref (1)	
	>0-4	11·39 (6·92-18·76)	11·14 (6·58-18·86)	0·12	6·08 (1·55-23·77)	0·40	6·18 (3·44-11·08)	0·76
	>4	11·95 (7·02-20·34)	11·78 (7·30-19·0)	0·70	27·53 (5·02-151·08)	0·36	10·34 (6·17-17·30)	0·08
**Doxycycline**								
	0	Ref (1)	Ref (1)		Ref (1)		Ref (1)	
	>0-20	6·19 (4·42-8·66)	2·23 (1·42-3·52)	<0·001	-	0·98	1·26 (0·38-4·17)	0·01
	>20	10·10 (6·45-15·82)	5·54 (3·02-10·16)	0·12	4·34 (045-41·70)	0·47	8·74 (3·00-25·43)	0·81
**UTI drugs**								
	0	Ref (1)	Ref (1)		Ref (1)		Ref (1)	
	>0-5	2·50 (1·44-4·35)	1·31 (0·95-1·82)	0·05	0·38 (0·09-1·56)	0·02	2·25 (0·85-5·96)	0·85
	>5	2·84 (2·07-3·90)	1·76 (1·39-2·23)	0·02	1·43 (0·71-2·89)	0·08	1·75 (0·94-3·27)	0·18

Abbreviations: CI, confidence interval; DDD/3PYR, defined daily dose per person in the three-years study period rate; OR, unadjusted odds ratio; UTI, urinary tract infection.

[1] p-value (wald test) testing if the association between antimicrobial prescription and subsequent HIV diagnosis is significantly different from the association in MSM.

The overall likelihood ratio test (LRT) for transmission mode differences in antimicrobial drug consumption in the 3 years period prior to HIV diagnosis was significant (LRT<0·01) only for the following antimicrobials: macrolides, quinolones, doxycycline, acyclovir and azoles.

Supplementary table S7. Association between antimicrobial prescription in the 3 years before HIV diagnosis and the risk of being diagnosed with HIV infection according to the different cut-offs of antibiotic prescription for macrolides, quinolones and doxycycline (HIV vs. matched controls).

A) All patients

	MSM	HTX		IDU		Unknown	
**DDD/P3Y**	OR (95% CI)	OR (95% CI)	p-value[1]	OR (95% CI)	p-value[1]	OR (95% CI)	p-value[1]
**Macrolides**							
**0**	Ref (1)	Ref (1)		Ref (1)		Ref (1)	
**>0 (vs 0)**	4·09 (3·63-4·61)	2·44 (2·12-2·81)	<0·001	1·68 (1·16-2·43)	<0·001	2·89 (2·10-3·97)	0·05
**>10 (vs ≤10)**	4·25 (3·57-5·06)	3·36 (2·77-4·07)	0·07	1·59 (0·84-3·04)	<0·001	3·30 (2·13-5·11)	0·29
**> 20 (vs ≤20)**	4·90 (3·78-6·35)	3·96 (2·99-5·24)	0·27	1·94 (0·75-5·02)	0·07	4·27 (2·31-7·92)	0·69
**Quinolones**							
**0**	Ref (1)	Ref (1)		Ref (1)		Ref (1)	
**>0 (vs 0)**	6·07 (4·96-7·43)	2·99 (2·31-3·88)	<0·001	1·90 (0·89-4·05)	0·004	2·57 (1·50-4·41)	0·004
**>7 (vs ≤7)**	4·82 (3·57-6·50)	3·19 (2·20-4·63)	0·09	1·18 (0·28-5·09)	0·07	2·69 (1·34-5·40)	0·13
**>14 (vs ≤14)**	6·48 (3·87-10·85)	4·51 (2·43-8·38)	0·38	-	0·98	2·02 (0·45-9·08)	0·15
**Doxycycline**							
**0**	Ref (1)	Ref (1)		Ref (1)		Ref (1)	
**>0 (vs 0)**	7·32 (5·59-9·59)	2·95 (2·05-4·23)	<0·001	0·68 (0·09-5·13)	0·02	2·92 (1·37-6·13)	0·02
**>20 (vs ≤20)**	9·85 (6·29-15·42)	5·48 (2·99-10·04)	0·13	4·33 (0·45-41·66)	0·49	8·71 (2·99-25·36)	0·84
**>40 (vs ≤40)**	8·67 (4·41-17·04)	5·57 (2·55-12·16)	0·40	-	0·99	32·5 (6·31-167·51)	0·14

B) Only male patients

	MSM	HTX men	
**DDD/3PY**	OR (95% CI)	OR (95% CI)	p-value[1]
**Macrolides**			
**0**	Ref (1)	Ref (1)	
**>0 (vs 0)**	4·09 (3·63-4·61)	2·79 (2·31-3·37)	<0·001
**>10 (vs ≤10)**	4·23 (3·55-5·05)	4·00 (3·07-5·21)	0·73
**> 20 (vs ≤20)**	4·86 (3·75-6·31)	4·45 (2·98-6·64)	0·71
**Quinolones**			
**0**	Ref (1)	Ref (1)	
**>0 (vs 0)**	6·12 (5·00-7·50)	3·78 (2·75-5·21)	0·013
**>7 (vs ≤7)**	4·87 (3·61-6·57)	4·06 (2·59-6·34)	0·51
**>14 (vs ≤14)**	6·48 (3·87-10·85)	5·57 (2·78-11·15)	0·73
**Doxycycline**			
**0**	Ref (1)	Ref (1)	
**>0 (vs 0)**	7·32 (5·59-9·59)	4·09 (2·54-6·57)	0·04
**>20 (vs ≤20)**	9·85 (6·29-15·42)	3·79 (1·63-8·80)	0·05
**>40 (vs ≤40)**	8·67 (4·41-17·04)	4·64 (1·67-12·89)	0·32

Abbreviations: CI, confidence interval; DDD/3PYR, defined daily dose per person in the three-years study period rate; OR, unadjusted odds ratio; UTI, urinary tract infection drugs.

[1] p-value (wald test) testing if the association between antimicrobial prescription and subsequent HIV diagnosis is significantly different from the association in MSM.

Supplementary table S8. Association between antimicrobial prescription in the 3 years before HIV diagnosis and the risk of being diagnosed with HIV infection stratified by HIV subgroups (HIV vs. matched controls).

	VLHIV	LHIV		EHIV	
**DDD/P3Y**	OR (95% CI)	OR (95% CI)	p-value[1]	OR (95% CI)	p-value[1]
Beta-lactams					
**>0 vs 0**	2·47 (2·15-2·82)	1·81 (1·50-2·18)	<0·01	2·04 (1·82-2·28)	0·03
**>10 vs ≤ 10**	2·60 (2·27-2·98)	2·04 (1·67-2·49)	0·05	2·16 (1·92-2·43)	0·04
**>20 vs ≤ 20**	3·18 (2·71-3·72)	2·33 (1·81-2·99)	0·04	2·46 (2·11-2·87)	0·02
**>30 vs ≤ 30**	3·70 (3·05-4·50)	2·98 (2·18-4·05)	0·24	2·79 (2·28-3·40)	0·05
Macrolides					
**>0 (vs 0)**	3·77 (3·28-4·34)	2·29 (1·86-2·83)	<0·01	3·02 (2·67-3·41)	0·02
**>10 (vs ≤10)**	5·06 (4·21-6·07)	2·09 (1·49-2·94)	<0·01	3·14 (2·59-3·79)	<0·01
**> 20 (vs ≤20)**	6·13 (4·77-7·89)	2·17 (1·24-3·78)	<0·01	3·43 (2·57-4·58)	<0·01
Acyclovir					
**>0 (vs 0)**	12·14 (9·76-15·09)	6·01 (4·30-8·41)	<0·01	4·85 (3·92-6·01)	<0·01
**>2 (vs ≤2)**	14·82 (11·60-18·95)	7·24 (4·96-10·56)	<0·01	4·33 (3·37-5·58)	<0·01
**>4 (vs ≤ 4)**	17·13 (13·23-22·18)	8·17 (5·52-12·10)	<0·01	3·97 (3·01-5·23)	<0·01
Azoles					
**>0 (vs 0)**	6·07 (4·99-7·40)	1·94 (1·36-2·78)	<0·01	1·97 (1·55-2·49)	<0·01
**>3 (vs ≤3)**	9·24 (7·29-11·71)	2·04 (1·22-3·44)	<0·01	2·44 (1·76-3·37)	<0·01
**>6 (vs ≤ 6)**	9·70 (7·50-12·55)	2·35 (1·35-4·09)	<0·01	2·57 (1·79-3·68)	<0·01
Quinolones					
**>0 (vs 0)**	3·30 (2·55-4·27)	2·98 (2·02-4·40)	0·67	5·42 (4·41-6·67)	<0·01
**>7 (vs ≤7)**	3·39 (2·37-4·84)	2·77 (1·59-4·81)	0·55	4·51 (3·28-6·19)	0·24
**>14 (vs ≤14)**	4·04 (2·15-7·60)	4·99 (1·93-12·90)	0·72	5·39 (3·14-9·24)	0·50
Nystatin					
**>0 (vs 0)**	18·42 (13·01-26·09)	3·79 (1·78-8·08)	<0·01	6·66 (4·31-10·27)	<0·01
**>4 (vs ≤4)**	22·86 (14·15-36·94)	4·99 (1·93-12·90)	<0·01	6·74 (3·53-12·85)	<0·01
**>8 (vs ≤8)**	28·36 (13·89-57·90)	4·88 (1·29-18·38)	0·02	13·0 (4·19-40·31)	0·25
Doxycycline					
**>0 (vs 0)**	3·48 (2·42-5·02)	3·48 (2·14-5·66)	0·99	6·24 (4·70-8·28)	0·01
**>20 (vs ≤20)**	4·84 (2·53-9·22)	8·67 (3·89-19·29)	0·27	10·01 (6·34-15·80)	0·07
**>40 (vs ≤40)**	7·94 (3·75-16·82)	8·67 (2·45-30·71)	0·91	8·05 (4·03-16·07)	0·98
UTI drugs					
**>0 (vs 0)**	1·75 (1·37-2·23)	1·93 (1·35-2·75)	0·65	1·79 (1·41-2·27)	0·89
**> 5 (vs ≤5)**	1·93 (1·46-2·55)	1·68 (1·10-2·58)	0·59	2·11 (1·61-2·76)	0·67
**>10 (vs ≤10)**	2·02 (1·43-2·85)	1·37 (0·76-2·47)	0·27	2·40 (1·68-3·41)	0·49

Abbreviations: CI, confidence interval; DDD/3PYR, defined daily dose per person in the three-years study period rate; EHIV, earlier HIV diagnosis; LHIV, late HIV diagnosis; OR, unadjusted odds ratio; UTI, urinary tract infection; VLHIV, very late HIV diagnosis.

[1] p-value (wald test) testing if the association between antimicrobial prescription and subsequent HIV diagnosis is significantly different from the association in VLHIV patients.

The overall likelihood ratio test (LRT) for differences according to HIV infection stage at diagnosis in antimicrobial drug consumption in the 3 years period prior to HIV diagnosis was significant (LRT<0·01) only for the following antimicrobials: macrolides, acyclovir, azoles and nystatin.
